# c.620C>T mutation in *GATA4* is associated with congenital heart disease in South India

**DOI:** 10.1186/s12881-015-0152-7

**Published:** 2015-02-18

**Authors:** Saidulu Mattapally, Sheikh Nizamuddin, Kona Samba Murthy, Kumarasamy Thangaraj, Sanjay K Banerjee

**Affiliations:** Division of Pharmacology, CSIR-Indian Institute of Chemical Technology, Uppal Road, Hyderabad, 500 007 India; Innova Children’s Heart Hospital, Tarnaka, Hyderabad, 500017 India; CSIR-Centre for Cellular and Molecular Biology, Uppal Road, Hyderabad, 500 007 India; Current Address: Drug Discovery Research Center, Translational Health Science and Technology Institute (THSTI), Gurgaon, HR-122016 Haryana India

**Keywords:** Congenital heart disease, *GATA4*, Mutation, South Indian patients, ASD, TOF, VSD

## Abstract

**Background:**

Congenital heart diseases (CHDs) usually refer to abnormalities in the structure and/or function of the heart that arise before birth. *GATA4* plays an important role in embryonic heart development, hence the aim of this study was to find the association of *GATA4* mutations with CHD among the south Indian CHD patients.

**Method:**

*GATA4* gene was sequenced in 100 CHD patients (ASD, VSD, TOF and SV) and 200 controls. Functional significance of the observed *GATA4* mutations was analyzed using PolyPhen, SIFT, PMut, Plink, Haploview, ESE finder 3.0 and CONSITE.

**Results:**

We observed a total of 19 mutations, of which, one was in 5′ UTR, 10 in intronic regions, 3 in coding regions and 5 in 3′ UTR. Of the above mutations, one was associated with Atrial Septal Defect (ASD), two were found to be associated with Tetralogy of Fallot (TOF) and three (rs804280, rs4841587 and rs4841588) were strongly associated with Ventricular Septal Defect (VSD). Interestingly, one promoter mutation (−490 to 100 bp) i.e., 620 C>T (rs61277615, p-value = 0.008514), one splice junction mutation (G>A rs73203482; p-value = 9.6e-3, OR = 6.508) and one intronic mutation rs4841587 (p-value = 4.6e-3, OR = 4.758) were the most significant findings of this study. *In silico* analysis also proves that some of the mutations reported above are pathogenic.

**Conclusion:**

The present study found that *GATA4* genetic variations are associated with ASD, TOF and VSD in South Indian patients. *In silico* analysis provides further evidence that some of the observed mutations are pathogenic.

**Electronic supplementary material:**

The online version of this article (doi:10.1186/s12881-015-0152-7) contains supplementary material, which is available to authorized users.

## Background

Congenital heart disease (CHD) usually refers to abnormalities in the structure and/or function of the heart that arise before birth. It has been estimated that about 1% and 6-8% of the newborns are affected with CHD in United States and worldwide, respectively [1]. Most of the studies conducted earlier reported that CHD has multi-factorial etiology, but recent studies indicate monogenic etiologies for a subset of CHDs [2-4]. Mutations in genes encoding transcription factor were found to cause nonsyndromic, human congenital heart disease. The knockout and transgenic mouse studies demonstrated that transcription factors play an important role during heart development. Approximately, more than 1,700 genes have been reported to be involved in the murine heart development [5]. GATA binding protein 4 (*GATA4*), NK2 homeobox 5 (*NKX2.5*), T-box 5 (*TBX5),* Zic family member *3 (ZIC3),* myosin heavy chain 6 *(MYH6)* and *NOTCH* are some of the transcription factors, which play crucial role in heart development [6].

Several familial studies reported that mutations in *GATA4* may cause different kind of CHD i.e., atrial septal defect (ASD), ventricular septal defect (VSD), tetralogy of fallot (TOF) and pulmonary stenosis (PS) [7-9]. Among all transcription factors, *GATA4* is the most studied gene in different populations [9-13]. *GATA4* has been mapped on 8p23.1 and has seven exons, which code for 442 amino acids and act as zinc finger transcription factor. *GATA4* plays an important role in embryonic heart development such as proliferation of cardiomyocytes, endocardial cushion formation, development of right ventricle and septation of the outflow tract. *GATA4* binds to the consensus sequence of the ANF promoter and interacts with other transcriptional factors such as; *NKX 2.5* and *TBX5* [14]. Subsequent studies have explained that *TBX5*, *NKX2-5* and *GATA4* interact during embryonic development and are involved in the regulation of heart developmental processes [6,15,16]. *GATA4* knockout mice produce phenotypes like ventral morphogenesis and heart tube formation [5].

ASD and VSD are the commonest types of CHD and account for 50% of all cases of CHD. If proper precautionary measures are not taken, these defects result in ventricular dilation and heart failure, and ultimately cause a decrease in life expectancy [17,18]. VSD and ASD are defects in the ventricular and atrial septum, the wall dividing the left and right ventricles, and atrium of the heart, respectively. TOF is a congenital heart defect, which is classically understood to involve in four anatomical abnormalities of the heart i.e. right ventricular hypertrophy, ventricular septal defects, pulmonic stenosis (PS) and overriding aorta of the heart. ASD, VSD and TOF are common form of congenital heart disease (CHD) in India and other countries. Compared to other familial CHDs, the prevalence of ASD and VSD is significantly higher [19]. *GATA4* has been identified as causative factors of familial ASD and VSD, and found to play a predominant role in the pathogenesis of both atrial and ventricular septal defects [7,20,21]. However, segregation of VSD and TOF with the *GATA4* mutation needs to be explored.

CHD is the leading cause of infant morbidity and mortality in India. Congenital heart diseases are found in 8–10 of every 1,000 live births [22] and nearly 1,80,000 children are born with CHD each year in India [23]. Of these, nearly 60,000 to 90,000 suffer from critical CHD requiring early intervention [23]. Because of higher rate of consanguineous marriages, frequency of congenital heart disease is very high in Andhra Pradesh state of southern India, compared to rest of India [24]. In spite of higher number of CHD in India, there are very few studies to find a link between *GATA4* mutation and congenital heart disease. Only two studies with CHD patients were conducted to find the association between few selected *GATA4* SNP with CHD, however, their data was not significant [25,26]. Therefore, in the present study we have sequenced the complete coding region including exon-intron boundaries of *GATA4* to elucidate the role of *GATA4* in the etiology of CHD in South Indian patients.

## Methods

### Clinical evaluation of CHD

Babies (including pre-mature infants), who were attending Innova Children’s Heart Hospital, Hyderabad were screened for CHD according to a pre-designed protocol, that include 2D echocardiography, color doppler and ECG. Of these different phenotypes, four different types of CHDs were considered for the present investigation; they are: atrial septal defect (ASD), ventricular septal defect (VSD), tetralogy of fallot (TOF), and single ventricle (SV).

### Patient and the control samples

A total of 100 patients, who fulfilled the criteria of CHD were selected for the study. Two hundred individuals, who had no CHD or family history of CHD or any heart disease, were also included in this study as control. The case samples (CHD) were drawn from Innova Children’s Heart Hospital, Hyderabad; while ethnically matched control samples were collected from Innova and other hospital in Hyderabad. Informed written consent was obtained from the parents of CHD patients and the control subjects prior to the collection of blood sample. In addition, we also took consent for ‘publication of individual patient data’ for all those individuals included in the manuscript (i.e.. all of those indicated in the family trees)”. All patient samples were collected from specialized hospital and they were belongs to same ethnic group (Dravidian). This study was conducted with prior approval of Institutional Ethical Committee of the Innova Children’s Heart Hospital, Hyderabad (IEC/IRB No. 001/2010) and IICT, Hyderabad.

### DNA isolation and sequencing

We collected about 2.0 ml of peripheral blood samples from all the patients and controls in EDTA-coated vacutainer and genomic DNA was isolated according to the protocol of Sambrook et al. 1989 [27]. *GATA4* sequence from the ENSEMBL (ID: ENSG00000136574; www.ensembl.org) was used to design primer employing primer 3 software (http://frodo.wi.mit.edu/) and was synthesized commercially (Eurofins, India). Detailed sequences of all primers used in this study have been summarized in Additional file 1: Table S1. The amplification reactions contained 40 ng of DNA, 10 mM dNTP mix, 10X PCR buffer, 2 U of Taq polymerase (Takara, catalogue no:R001B) and 5 pM of both the primers. PCRs were carried out using ABI GeneAmp PCR System 9700 (Forster city, USA). Amplification conditions used were as follows; an initial denaturation at 95°C for 5 min., followed by 35 cycles of denaturartion at 94°C for 1 min, annealing at 55-64°C for 30 sec (Additional file 1: Table S1) and at 72°C for 1 min. A final extension was carried out at 72°C for 10 min. PCR products were cleaned up using Exo-SAP-IT (USB, Affymetrix, USA) and 1.0 μl of the purified products were directly used as templates for sequencing using di-deoxy chain terminator cycle sequencing protocol (BigDye V3.1, Applied Biosystems, Forster city, USA) [28]. The extended products were purified by ethanol precipitation and run in an ABI 3730 Automated DNA Analyzer (Applied Biosystems, Forster city, USA). Sequencing was carried out using both forward and reverse primer independently.

### Mutation analysis

The raw sequence data were analyzed and carefully edited using the Sequence Analysis Software. The edited sequences were assembled with reference sequence using DNA Star and Auto Assembler software (Applied Biosystems, USA). All the variant sites, compared to the reference sequence, were noted down. Genetic Association, Hardy–Weinberg equilibrium and Chi-square test were computed by using plink software [29]. Pathogenic potential of identified missense mutations from CHD patients was predicted by three different softwares. The prediction of PolyPhen software (www.tux.embl-heidelberg.de/ramensky/polyphen.cgi) was determined based on sequence comparison between homologous proteins. Profile scores position-specific independent counts (PSIC) were generated for the allelic variants. A PSIC score of the variant with more than 2 indicates damaging effect, scores between 1.5 and 2 indicate possibly damaging effects, score 1.5 indicates that the variant is probably damaging and score less than 1.5 indicates that the variant is benign. PMut, software for mutation prediction, is pathogenicity index ranging from 0 to 1. Indices greater than 0.5 indicate pathological mutation while less than 0.5 indicate neutral. SIFT is another sequence homology-based tool that identifies intolerant from tolerant amino acid substitutions. This software predicts whether an amino acid substitution at a particular position in a defined protein will have a phenotypic effect or not. For prediction of mutation effect on splicing of intron, we used ESE finder 3.0 [30]. This software finds the changes in the binding of splicing enhancers. ‘R’ packages were used for generation of plot. Promoter mutation’s function was predicted by CONSITE software, which finds the difference of transcription factor binding into wild type and mutant genomic sequences.

### Effect of 3’ UTR region mutations on microRNA-target interactions

The interaction of 3′UTR with microRNA was determined by a bioinformatics tool as described by Kertesz et al. 2007 [31]. This bioinformatics tool finds microRNA-target interactions by an energy score, ΔΔG that is equal to the difference between the energy gained by binding of the microRNA to the target, and dGopen, the energy required to make the target region accessible for microRNA binding. dGduplex is the binding free energy of the microRNA-target duplex structure in which the microRNA and target are paired according to pairing constraints imposed by the seed. ΔΔG is an energetic score, the lower (more negative) the value, the stronger the binding of the microRNA to the given site is expected to be. As a rough rule of thumb, sites having ΔΔG values below −10 are likely to be functional in endogenous microRNA expression levels.

### Statistical analysis

Statistical analysis was performed with Plink software [29]. For checking markers, whether they are in hardy Weinberg equilibrium, we used cut off p-value 0.01; and for association analysis we used same cut off p-value (0.01). Haploview was used to find blocks in data [32]. For finding the blocks, we considered r2 value and LOD score. Further, we used PHASE software for making haplotype for these blocks [33] and did association analysis with R basic packages (R version 3.0.2, 2013) [34].

## Results

### Clinical evaluation

In the present study, we have analyzed a total of 100 CHD patients. The percentages of CHD patients belonging to different categories were as follows; ASD: 33%, VSD: 32%, TOF: 32%, and SV: 3%. Age of all CHD patients ranged from 0.35 to 10.79 years. However, maximum number of CHD patients taking part in this study were of <5 years (Table 1).

**Table 1:** **Clinical classification of CHD patients studied**

**S. no**	**Type of CHD**	**Patients (n = 100)**	**Age**				
			**>1 year**	**1-5 years**	**5-10 years**	**10-15 years**	**15-20 years**
1	ASD	33(33%)	2	18	8	3	2
2	VSD	32(32%)	11	17	3	1	-
3	TOF	32(32%)	12	16	3	1	-
4	SV	3(3%)	2	1	-	-	-
	Total	100	27	52	14	5	2

### Mutation analysis in *GATA4* gene

**Figure 1:**
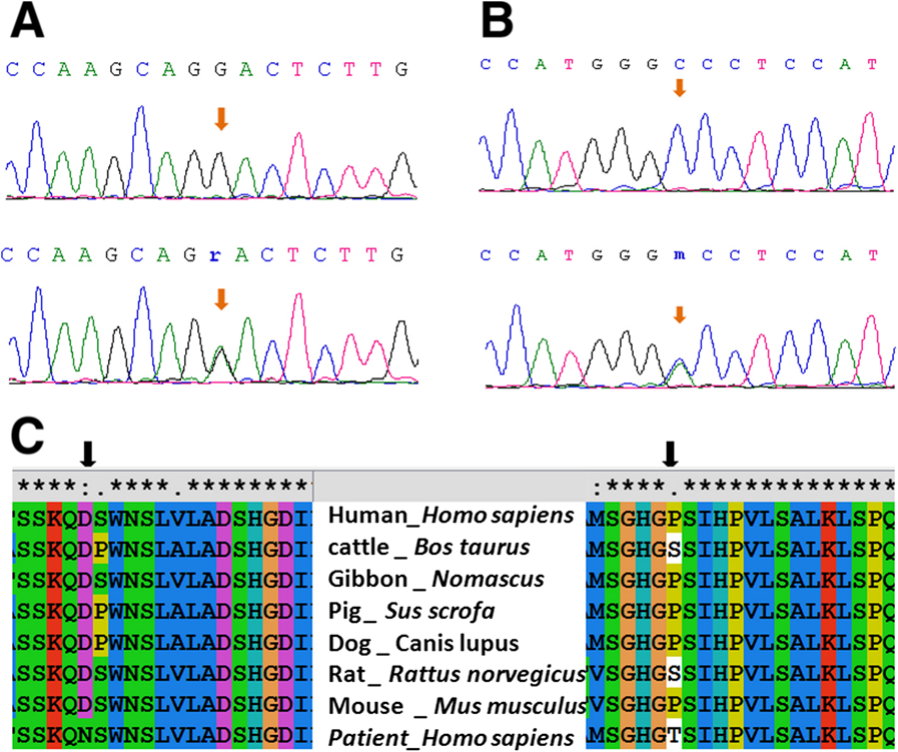
**DNA sequencing electropherogram showing wild type (upper panel) and heterozygous (G → A) mutation (lower panel) in** ***GATA4*** **coding region (arrows). A**. Mutation Asp425Asn, **B**. Mutation Pro394Thr, **C**. Multiple amino acid sequence alignment of different species shows the conservation of the mutated amino acid residue (amino acid marked) across species.

**Figure 2:**
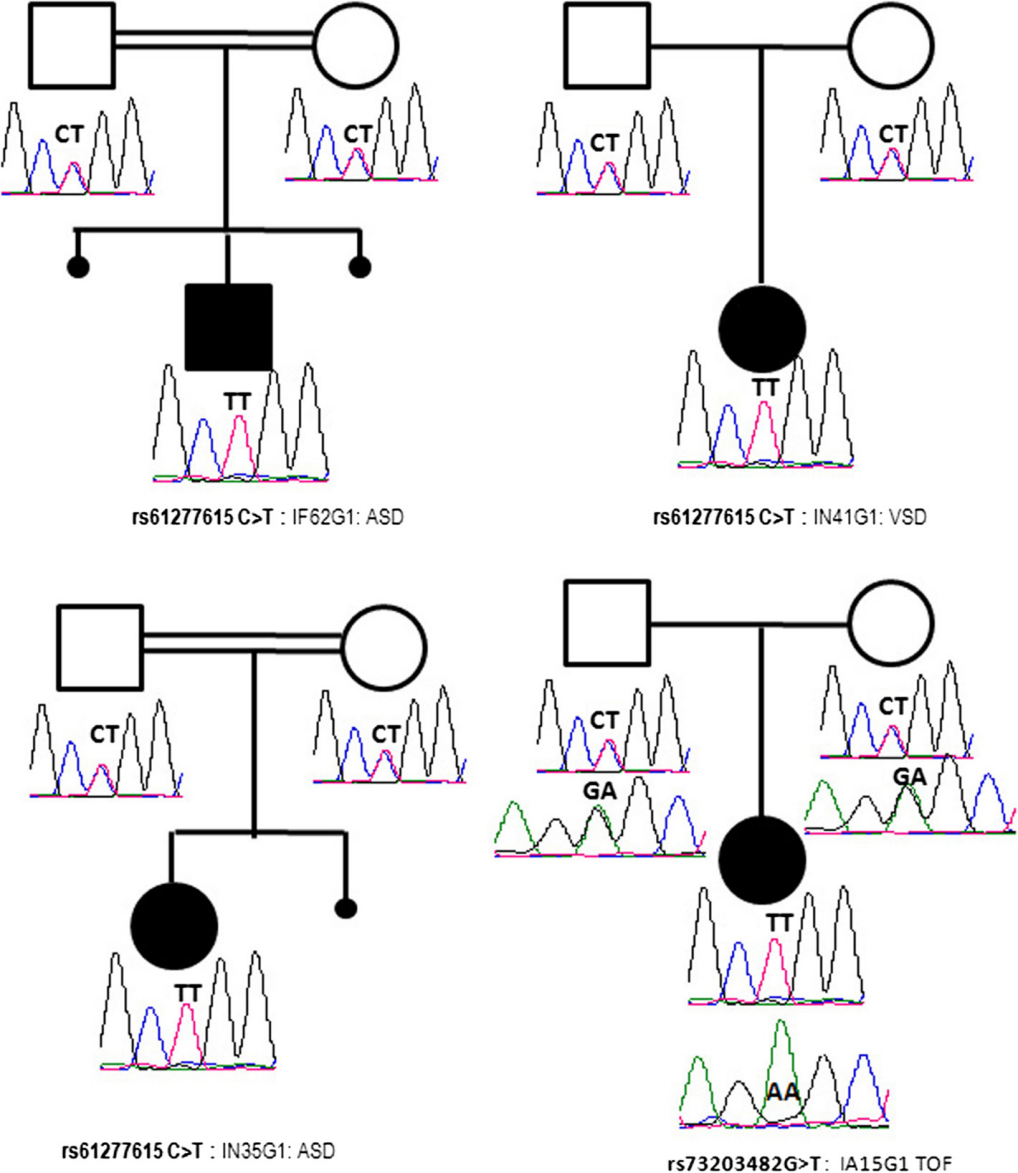
**DNA sequencing electropherogram of promoter mutation (rs61277615) and a splice junction mutation (rs73203482) showing heterozygous condition in parents and homozygous condition and the patients.**

We investigated the genomic DNA of CHD patients for variations in the entire coding regions, exon-intron boundaries, and untranslated regions (3′ UTR and 5′ UTR) of *GATA4* gene. Our analysis revealed a total of 19 mutations, of which 1 each in promoter and splicing regions, 9 were in intronic regions, 3 were in exonic regions (2 missense and one synonymous) and 5 were in 3′ UTR (Tables 2 and 3). *In silico* analysis showed that one missense mutation was conserved and predicted to be pathogenic (Figure 1). Figure 2 shows sequence electropherogram of two mutations, which were evidenced in promoter and splicing regions. We evidenced the presence of homozygous mutation (rs61277615) at the promoter region in two ASD and one VSD patients, and (rs73203482) at the splice site in one TOF patients. We have collected parent’s samples for those patients who have either rs61277615 or rs73203482 variations in *GATA4* gene. Analysis of parents’ samples revealed that parents of all the three patients had heterozygous mutation (Figure 2).

**Table 2:** **Detailed description of** ***GATA4*** **mutations identified in this study**

**S. no.**	**dbSNP**	**Exon/intron**	**Coordinate position**	**Nucleotide variation**	**Mutation type**	**A.A change**	**Frequency of mutation**				
							**ASD**	**VSD**	**TOF**	**SV**	**Control**
							**33**	**32**	**32**	**3**	**200**
1	rs61277615	Exon1	11561728	C>T	5′UTR	-	0.06061	0.03125	0.03125	0	0.0125
2	rs73203482	Intron1	11561818	G>A	Splice junction	-	0.0303	0.03125	0.04688	0	0.0075
3	CM051488	Exon3	11606451	G>A	Synonymous	G214G	0.04545	0	0	0	0.0125
4	rs804280	Intron5	11612698	A>C	Intronic	-	0	0.07812	0.0625	0	0.0125
5	rs2645457	Intron5	11614112	T>G	Intronic		0.01515	0.01562	0.01562	0	0.005
6	rs4841587	Intron5	11614175	G>T	Intronic	-	0.0303	0.07812	0.03125	0	0.0175
7	rs4841588	Intron5	11614225	G>T	Intronic	-	0.01515	0.1562	0.0625	0	0.02
8	rs111272281	Intron5	11614264	A>del	Intronic	-	0	0.0625	0.03125	0	0.005
9	rs3729853	Intron5	11614316	C>T	Intronic	-	0.0303	0.2188	0.07812	0	0.055
10	rs3729854	Intron5	11614329	C>T	Intronic	-	0.01515	0.04688	0.04688	0	0.01
11	rs142395583	Intron5	11614337	T>A	Intronic	-	0	0.01562	0.01562	0	0.0125
12	rs745379	Intron6	11615695	A>G	Intronic	-	0.04545	0.1094	0.1719	0.16	0.01
13	rs200319078	Exon7	11615835	C>A	Missence	P394T	0.01515	0	0	0	0.005
14	rs56208331	Exon7	11615928	G>A	Missence	D425N	0	0.01562	0	0	0.0025
15	rs884662	Exon7	11616501	T>C	3′UTR	-	0	0.01562	0.01562	0	0.0125
16	rs904018	Exon7	11616516	C>T	3′UTR	-	0.04545	0.01562	0.03125	0	0.0225
17	rs12825	Exon7	11616547	C>G	3′UTR	-	0.01515	0.09375	0.1094	0	0.0825
18	rs12458	Exon7	11617240	A>T	3′UTR	-	0	0	0	0	0.01
19	rs3203358	Exon7	11617505	C>G	3′UTR	-	0.01515	0.01562	0.01562	0	0.0125

**Table 3:** **The allelic distributions of** ***GATA4*** **polymorphism among patients and controls**

**dbSNP**	**Control allele (n = 400)**	**ASD allele (n = 66)**	**VSD allele (n = 64)**	**TOF allele (n = 64)**	**Control vs. ASD**		**Control vs. VSD**		**Control vs. TOF**	
					**p-value**	**OR (95% CI)**	**p-value**	**OR (95% CI)**	**p-value**	**OR (95% CI)**
rs61277615	5(0.0125)	4(0.06061)	2(0.03125)	2(0.03125)	**0.008514**	**5.0968(1.3322 - 19.4998)**	0.2532	2.54849(0.4838 - 13.4239)	0.2532	2.54849(0.4838 - 13.4239)
rs73203482	3(0.0075)	2(0.0303)	2(0.03125)	3(0.04688)	0.09573	4.1354(0.6777 - 25.2338)	0.08752	4.2688(0.6992 - 26.0617)	0.009632	**6.5082(1.2843 - 32.9812)**
rs804280	5(0.0125)	0	5(0.07812)	4(0.0625)	-	-	**0.0007886**	**6.6949(1.8812 - 23.8262)**	**0.007083**	**5.2667(1.3755 - 20.1654)**
rs4841587	7(0.0175)	2(0.0303)	5(0.07812)	2(0.03125)	0.4838	1.7545(0.3565 - 8.6342)	**0.004553**	**4.7579(1.4622 - 15.4814)**	0.459	1.8111(0.3678 - 8.9182)
rs4841588	8(0.02)	1(0.01515)	10(0.1562)	4(0.0625)	0.7909	0.7538(0.0927 - 6.1274)	**0.0000001597**	**9.0741(3.4321 - 23.9913)**	0.04671	3.2667(0.9542 - 11.1831)

### Genetic studies

To find out association between genetic markers and CHD, we initially analyzed the effect of SNPs independently and then analyzed the haplotype level. We classified the samples on the basis of phenotype: ASD, VSD, TOF, and SV and removed 6 markers, which were not following Hardy-Weinberg equilibrium in ethnically matched control samples. These markers were rs12825 (HWE p-value = 1.296e-7), rs745379 (HWE p-value = 1.894e-5), rs3203358 (HWE p-value = 9.47e-5), rs3729853 (HWE p-value = 1.855e-15), rs111272281 (HWE p-value = 2.506e-3) and rs904018 (HWE p-value = 2.35e-3) (Additional file 1: Figure S1).

**Figure 3:**
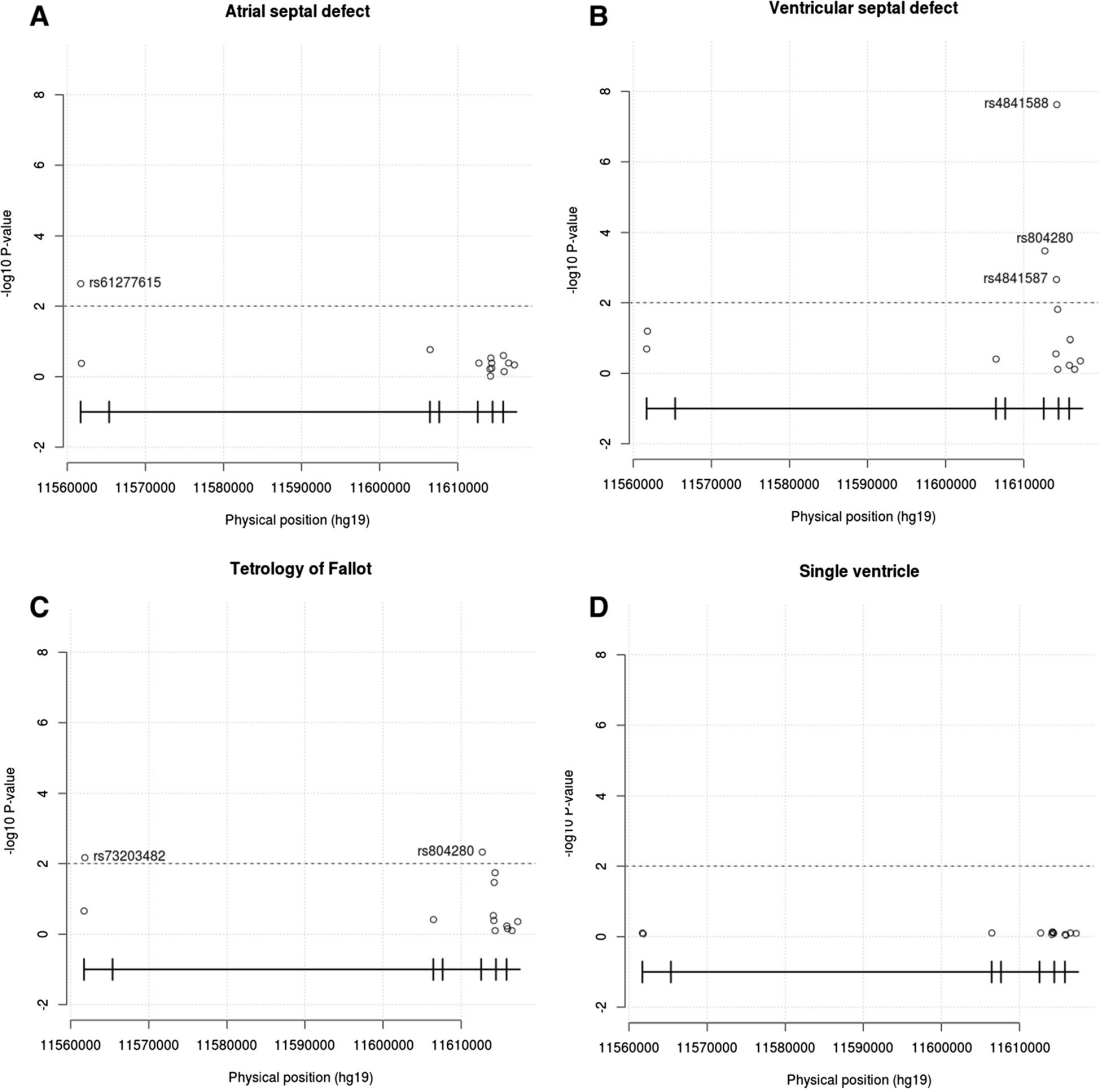
**Chi-square P-value of variations found in** ***GATA4*** **of all types of CHD. A**. atrial septal defect (ASD), **B**. ventricular septal defect (VSD), **C**. tetralogy of fallot (TOF) and **D**. single ventricle (SV).

Further, we performed Chi-square analysis for finding the statistical significance and identified one 5′ UTR (promoter region −490 to 100 bp) mutation (rs61277615, p-value = 0.008514) in ASD. In TOF, rs73203482 (p-value = 9.6e-3, OR = 6.508) and rs804280 (p-value = 7.08e-3, OR = 5.267) were found to be associated with the disease. In case of VSD, 3 mutations were found to have strong association with the disease; they were rs804280 (p-value = 7.9e-4, OR = 6.695), rs4841587 (p-value = 4.6e-3, OR = 4.758) and rs4841588 (p-value = 1.6e-7, OR = 9.074). In case of SV, we did not find any variation associated with the CHD phenotype (Figure 3, Table 4).

**Table 4:** **Statistical data of** ***GATA4*** **mutations and their association with ASD, VSD, TOF and SV**

**S. no.**	**dbSNP**	**Observed heterozygosity**	**Expected heterozygosity**	**Hardy Weinberg P-value**	**P-value**			
					**ASD**	**VSD**	**TOF**	**SV**
1	rs61277615	0.04	0.0392	1	0.008514	0.2532	0.2532	0.7829
2	rs73203482	0.015	0.01489	1	0.09573	0.08752	0.009632	0.8314
3	CM051488	0.01	0.00995	1	0.003122	0.5708	0.5708	0.8622
4	rs804280	0.025	0.02469	1	0.3611	0.0007886	0.007083	0.7829
5	rs2645457	0.01	0.00995	1	0.3394	0.3248	0.3248	0.8622
6	rs4841587	0.025	0.03439	0.05197	0.4838	0.004553	0.459	0.7438
7	rs4841588	0.03	0.0392	0.06886	0.7909	1.597e-07	0.04671	0.7264
8	rs111272281	0	0.00995	**0.002506**	-	-	-	-
9	rs3729853	0.01	0.104	**1.855e-15**	-	-	-	-
10	rs3729854	0.01	0.0198	0.01502	0.7067	0.02464	0.02464	0.8056
11	rs142395583	0.025	0.02469	1	0.3611	0.8372	0.8372	0.7829
12	rs745379	0	0.0198	**1.894e-05**	-	-	-	-
13	rs200319078	0.01	0.00995	1	0.3394	0.5708	0.5708	0.8622
14	rs56208331	0.005	0.004987	1	0.6843	0.1367	0.6888	0.9024
15	rs884662	0.015	0.02469	0.02497	0.3611	0.8372	0.8372	0.7829
6	rs904018	0.025	0.04399	**0.002346**	-		-	-
17	rs12825	0.075	0.1514	**1.296e-07**	-		-	-
18	rs12458	0.01	0.0198	0.01502	0.4145	0.4217	0.4217	0.0606
19	rs3203358	0.005	0.02469	**9.47e-05**	-		-	-

Bold indicates: The SNP which is not in HW-equilibrium and P-value < 0.01.

### *In silico* analysis

**Figure 4:**
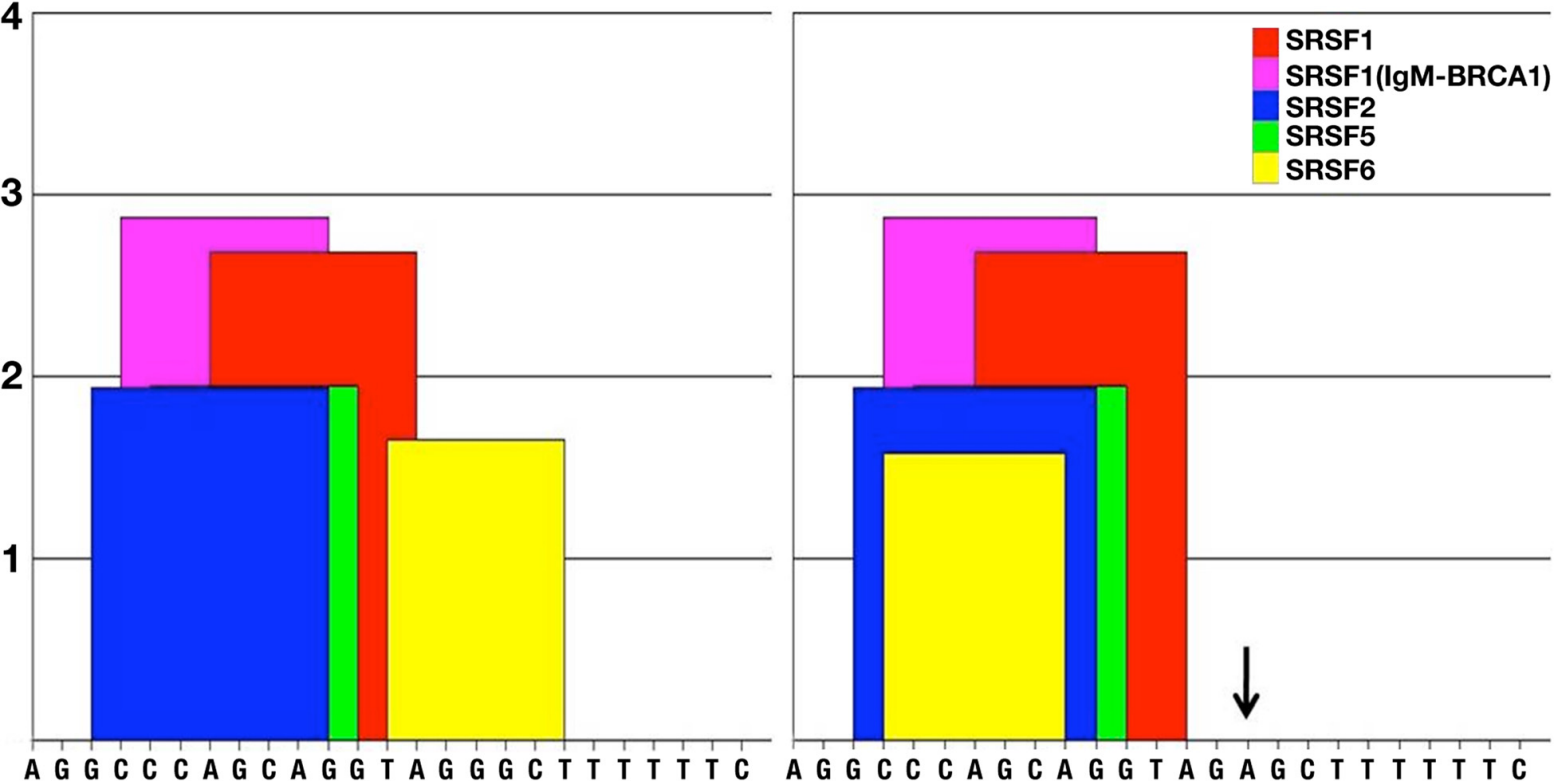
**Prediction for binding of splicing enhancer: an outcome of rs73203482 mutation.**

In coding region, we found a total of two missense mutations and one synonymous mutation in CHD patients that include Pro394Thr, Asp425Asn and Gly214Gly. Multiple alignments of *GATA4* amino acid sequences from human, cattle, gibbon, pig, dog, rat and mouse found that asparagine at 425 position (Figure 1B) is highly conserved during evolution. But other missense mutation i.e., Pro394Thr is not conserved. To understand the functional significance of missense mutations, we performed bioinformatics analysis with all of identified two missense mutations using PolyPhen-2, PMut and SIFT softwares. Results of all the three analyses strongly indicate that Asp425Asn mutation was pathogenic (Table 5). We analyzed functional significance of splice junction mutation (rs73203482) by ESE finder 3.0 and found that this mutation is present near splice site of exon 1 (ENST00000335135) and intron 1. The data has shown the effect of binding of SFRS6, a splicing factor to the splicing region (Figure 4). This splicing factor has a role in site selection in alternative splicing. This also explains that the binding of splice enhancer will be affected in the presence of this splice mutation. We computed the functional significance of promoter mutation (promoter region −490 to 100 bp, rs61277615) and found the transcription factor binding in the wild-type and mutant promoter region by CONSITE software. Our data indicated that mutant sequence strongly binds with the transcription factor Myf1 with score of 7.360 in comparison to wild type sequence. The score with more than 7.0 represents the stronger binding of transcription factor with the DNA sequence. Except Myf1, binding of other transcription factors for both wild and mutant sequence is the same (Additional file 1: Table S2).

**Table 5:** **Functional significance of mutation prediction done by using PolyPhen-2, PMut and SIFT**

	**P394T**		**D425N**	
**Name of software**	**Prediction**	**Score**	**Prediction**	**Score**
PolyPhen-2	Benign	0.025	Possibly damaging	0.972
PMut	Neutral	0.4499	Pathological	0.6236
SIFT	Tolerated	0.55	Not Tolerated	0.24

### Haplotype analysis

**Figure 5:**
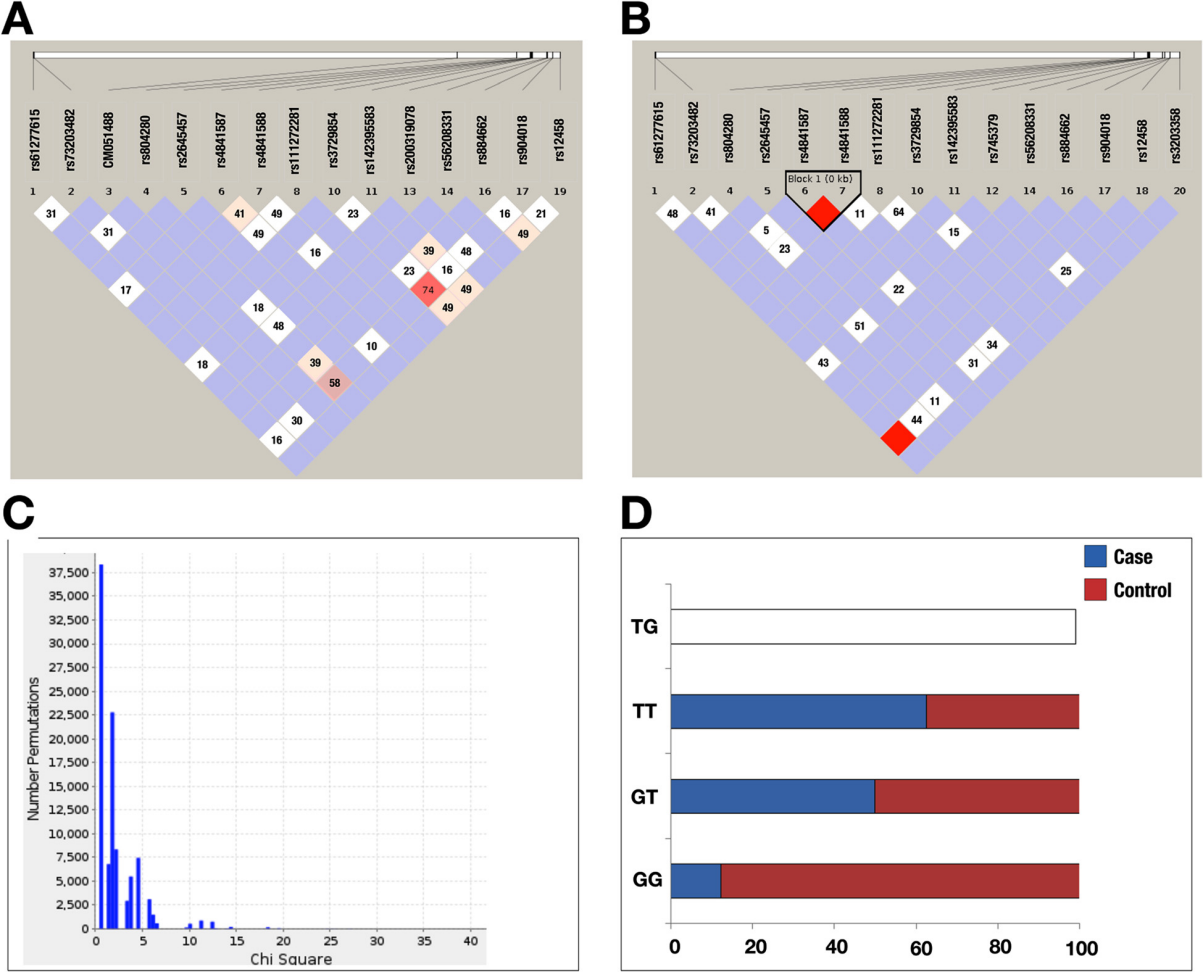
**Haplotype analysis. A**. Haplotype frequency distribution of control samples. **B**. Linkage disequilibrium in VSD samples. **C**. Permutation based analysis for haplotype association. **D**. Haplotype analysis of VSD samples and its allelic distributions in cases and controls.

Although SNPs have shown association with CHD, we further checked whether any haplotypes also show association with CHD. We did not find any block of associated markers in control samples (Figure 5A) but we found one LD block in VSD samples (Figure 5B). We also did not find any LD block in TOF samples (Additional file 1: Figure S2). We found that one LD block showed strong associations in VSD (p-value; GG: 1.906e-6, GT: 7e-4, TT: 6.01e-5) (Figure 5D, Additional file 1: Table S3). Same LD block also showed association in 10000 permutations (p-value; GG: 8e-4, GT: 2.9e-3, TT: 1.31e-2) (Figure 5C, Additional file 1: Table S4).

### Comparison of allele frequency with 1000 genome project data

**Figure 6:**
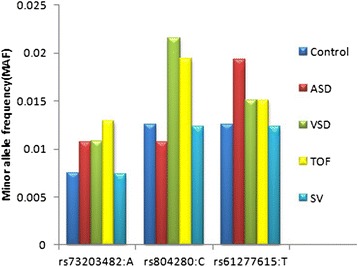
**Distribution of minor allele frequency of associated** ***GATA4*** **variations in ASD, VSD, TOF, SV, control sample.**

We compared the allele frequency of associated markers with 1000 genome project data. There was no significant difference in the allele frequency of control samples and Asian population samples of 1000 genome project (Additional file 1: Figure S3). While we observed differences between patient samples of VSD, TOF and ASD in comparison to control samples. Only three markers rs73203482, rs804280 and rs61277615 showed difference in distribution of allele frequency between disease samples and control samples (Figure 6).

### 3′ UTR mutations and their effect on microRNA-target interactions

Exon 7 of *GATA4* consists of 1,708 bp the majority (1,525 bp) being untranslated. We identified 5 dbSNPs in 3′-UTR region of *GATA4* in the study subjects; that include 2400T>C (rs884662), 2415T>C (rs904018), 2446C>G (rs12825), 3139A>T (rs12458) and 3404C>G (rs3203358). We did RNA folding studies for 1500 bp of 3′-UTR of *GATA4* gene that include all of the above SNPs independently using DNA Star software, but we did not find any change (data not shown). We then checked for the microRNA and 3′-UTR target interaction through a bioinformatics tool. We found interaction of 440 numbers of microRNAs with desired UTR sequence. We found change in free energy difference of microRNA binding between wild type and mutant UTR regions. The value of microRNA-target interactions, ΔΔG was found to be equal to the difference between the free energy gained by the binding of the microRNA to the target. As a rough rule of thumb, sites having ΔΔG values below −10 were found likely to be functional in endogenous microRNA expression levels. While the free energy change (ΔΔG) of wild-type UTR region was more than −10, it completely shifted to +10 value for all 3′UTR mutant (Additional file 1: Figure S4A and 4B).

## Discussion

Genetics and environmental factors play important role in the etiology of CHD. It has been shown that fetal heart development is regulated by a group of highly conserved transcription factors [35]. *GATA4* is one of the transcription factors that plays a major role in the regulation of several other cardiac proteins, including atrial natriuretic peptide, brain natriuretic peptide, cardiac troponin C, cardiac troponin I, muscarinic acetylcholine receptor, and slow myosin heavy chain. Small changes in the level of *GATA4* protein expression can dramatically influence cardiac development and embryonic survival [36]. *GATA4* has been identified in familial and sporadic cardiac septal defects. Although several studies from different countries have established the association of *GATA4* mutation with CHD [11,37], very few studies have been conducted in India. Therefore, we conducted this study to find genetic link with *GATA4* mutation and correlate genotype with phenotype from subjects with ASD, VSD, TOF and single ventricle.

Analysis of all the exons of *GATA4* revealed 19 mutations, out of which 2 were in promoter region, 9 were in intronic region, 3 were in coding region (that include 2 missense mutations and one synonymous mutation) and 5 were in 3′ UTR. The *GATA4* mutation frequencies from different studies in VSD were as follows; in Americans 1.67% (2/120) [10], Chinese 1.22% (1/82) [11], Americans 0.80% (5/628) [38], Germans 16.7% (1/6) [12], and Chinese 0.48% (1/210) [8] whereas in case of TOF the frequencies were; Americans 7.69% (2/26) [10] and Chinese 8.33% (1/12) [11]. Since, Dravidian (Indian) population has different genetic architecture [39,40], we observed high frequency of rs73203482 in TOF, rs804280 in VSD, rs61277615 in ASD in comparison to ASD, VSD, SV and control samples. The associations of rs804280 with VSD and TOF, rs73203482 with TOF and rs61277615 with ASD are interesting findings (Additional file 1: Figure S2). These markers may have major contribution in CHD disease in Dravidian population but need to be validated in large number of samples. *GATA4* is a hyper mutable protein according to previously published data [41]. Currently, approximately 111 non-synonymous mutations have been found in the *GATA4* protein, including the mutations identified in this study. We identified two missense mutations i.e., Pro394Thr, and Asp425Asn and one synonymous mutation Gly214Gly in the coding region of CHD patients. The amino acid that changes due to novel mutation is conserved throughout the species. All three softwares PolyPhen (score 0.97), Pmut (score 0.8625) and SIFT (score 0.27) showed that missense mutation Asp425Asn is probably damaging, pathological and not tolerated but other missense mutation Pro394Thr is benign, not pathological and tolerated. Novel mutation, Asp425Asn maps to the C-terminal domain of *GATA4*, and is important for the trans-activation of downstream targets [42].

We observed one mutation in *GATA4* gene promoter region (−490 to 100 bp). This exists in homozygous condition in; two ASD patients, one VSD patient and one TOF patient. Further analysis of this mutation in the parents of the above patients revealed the presence of heterozygous mutation. Due to high rate of consanguineous marriages among South Indian populations, this recessive mutation is much more common in the study populations, compared to other populations. We analyzed transcription factor binding with the wild and mutant type *GATA4* gene and found that one transcription factor strongly binds to the mutant but not with the wild type. We also found one mutation near the promoter region, specifically in the splicing region. Position of this mutation is interesting as it may affect the splicing of *GATA4* gene. Alternative first exons are located several kilobases upstream of the classic *GATA4* transcription initiation site suggesting that their expression is being driven from novel upstream promoters. Some of these first exons are conserved across species suggesting alternative promoter usage to be likely an important regulatory mechanism for controlling the tissue- and cell-specific expression of the *GATA4* gene in humans and other mammalian species. *GATA4* first exons are used alternatively and rarely in association with one another. The rs73203482, which is present near to splicing site of exon 1 (ENST00000335135) and in intron 1 has shown to affect binding of SFRS6, a splicing factor (Figure 4). This splicing factor has a crucial role in site selection in alternative splicing.

In the present study, we also observed several mutations at the 3′UTR region of *GATA4*. We evidenced five 3′ UTR mutations from patients with ASD, VSD and TOF. All the five mutations have been already reported by different investigators [43]. However, Kertesz et al. experimentally showed that mutations at 3′UTR substantially reduce microRNA-mediated translational repression, with effects comparable to those of mutations that disrupt sequence complementarity. They devise a bioinformatics tool for microRNA-target interaction that computes the difference between the free energy gained from the formation of the microRNA-target duplex and the energetic cost of un-pairing the target to make it accessible to the microRNA. We used the same software to study the functional role of the 3′UTR mutation. However, none of the study showed any functional study of these mutations for the pathogenesis of CHD. Here, we used bioinformatics tools to find out if these mutations in 3′UTR cause any alteration of *GATA4* function. The 3′-UTR of *GATA4* is relatively long and likely contains regulatory elements essential for the regulation and transport of the mRNA transcript [44]. Accumulating evidence suggests that the 3′-UTR of mRNA is involved in the control of nuclear transport, polyadenylation status, sub-cellular targeting as well as rates of translation and degradation of mRNA by altering RNA secondary structure. One of the reasons for alteration of RNA secondary structure is due to aberrant RNA folding [45]. To confirm the alteration of secondary structure of RNA due to mutations in 3′ UTR, we performed RNA folding studies, but we did not observe any changes in RNA folding between wild type and mutants (data not shown). We approached a parameter free model for miRNA–target interactions (Pita algorithm). Previously, this bioinformatics tool was used to find the difference of miRNA–target interactions among mutated gene [31]. Using this tool, we predicted the thermodynamic changes during miRNA–mRNA duplex formation. Our results showed that in wild *GATA4*, a pool of microRNA binds strongly with the mRNA. However, this binding affinity of miRNA was altered due to the presence of 3′UTR mutations. Our study strongly predicts that all 3′UTR *GATA4* mutations observed in CHD patients may alter the transcript level in diseased heart and affect the embryonic development of heart.

## Conclusion

Our study identified c.620C>T mutation in *GATA4* is associated with CHD in South India. Although this mutation reported in healthy individuals of other population. For the first time we are reporting its association with CHD in Indian population. Out of four phenotypes we studied, genetic variations were found to be associated with ASD (rs61277615), VSD (rs804280) and TOF (rs73203482, rs804280). c.620C>T either independently or combinly associated with CHD. Hence *GATA4* is an important marker for CHD in India, particularly South India.

## Additional file

Additional file 1: Table S1. List of primers used for amplification and sequencing of *GATA4*. **Table S2.** The list of transcription factors and their binding score with the wild type and mutant sequence. **Table S3.** Haplotype based association analysis of block 1 for VSD samples. **Table S4.** Permutation based analysis for haplotype association: 10000 permutations. **Figure S1.** Hardy-Weinberg equilibrium P-value. **Figure S2.** Linkage disequilibrium in TOF samples. **Figure S3.** Distribution of minor allele frequency of observed SNPs in control and 1000 genome project samples (ASN: Asian,CHB: Han Chinese in Bejing, China, CHS: Southern Han Chinese, JPT: Japanese in Tokyo, Japan,, EUR : European, AMR: American). **Figure S4.** A. Free energy change (ΔΔG) for Micro RNA binding with wild-type of 3′UTR region. B. Free energy change (ΔΔG) for Micro RNA binding with mutant of 3′UTR region.
