# Supplementary material for: c.620C>T mutation in GATA4 is associated with congenital heart disease in South India
Source: BMC Med Genet. 2015 Feb 18;16:7. doi: 10.1186/s12881-015-0152-7 (PMC4422155; doi:10.1186/s12881-015-0152-7)
Supplement: Additional file 1: Table S1. — List of primers used for amplification and sequencing of GATA4. Table S2. The list of transcription factors and their binding score with the wild type and mutant sequence. Table S3. Haplotype based association analysis of block 1 for VSD samples. Table S4. Permutation based analysis for haplotype association: 10000 permutations. Figure S1. Hardy-Weinberg equilibrium P-value. Figure S2. Linkage disequilibrium in TOF samples. Figure S3. Distribution of minor allele frequency of observed SNPs in control and 1000 genome project samples (ASN: Asian,CHB: Han Chinese in Bejing, China, CHS: Southern Han Chinese, JPT: Japanese in Tokyo, Japan,, EUR : European, AMR: American). Figure S4. A. Free energy change (ΔΔG) for Micro RNA binding with wild-type of 3′UTR region. B. Free energy change (ΔΔG) for Micro RNA binding with mutant of 3′UTR region. [file 12881_2015_152_MOESM1_ESM.pdf]

SUPPLEMENTAL MATERIAL

Supplementary Table 1. List of primers used for amplification and sequencing of *GATA4*.

S.No.	Primer	Primer Sequence	Annealing Temp	Product size (bp)
1	S1 GatA4_h	AGCTTTCCGCACAGTCCC	64	348
2	AS1 GatA4_h	CCCACTAGCTACCTCTCTACC		
3	S2 GatA4_h	AAATCCCCGTGGCGACTTCAT	61	399
4	AS2GatA4_h	CAGCATTGAGCAAAGGGCTCTA		
5	S3 GatA4_h	TCCGGAGTAAACAAGAGCCTAGA	60	494
6	AS3 GatA4_h	CTCCCGCCTGGCTCCATC		
7	S4 GatA4_h	GCCGACGGAGCCGCTTAC	64	432
8	AS4 GatA4_h	GAAGGCGTTGGTGGAAAAACAAGA		
9	S5 GatA4_h	GGCATTGTTTCTGTGCGCTCT	59	385
10	AS5 GatA4_h	CACGTAATCCCCGATGCACAC		
11	S6 GatA4_h	CTCATGCAGGGTCGTTAGGG	59	400
12	AS6 GatA4_h	GCAAAGTAGTTGAAAGCCCCTT		
13	S7 GatA4_h	CTTTCTCGCTGAGTTCCAGGG	63	387
14	AS7 GatA4_h	GAAAGGCCAGGGATGTCCGATG		
15	S8 GatA4_h	CCTCCGCAGATAAGGACCTC	62	428
16	AS8 GatA4_h	TAGCGCAGAGGGTAGCTCA		
17	S9 GatA4_h	GAGAGAAGTGCTCCTTGGTCC	59	450
18	AS9 GatA4_h	CCCAGTTGTTGTTCTGGAGTCATTA		
19	S10 GatA4_h	GATAGCAAAGAAGGAGGCCC	66	450
20	AS10 GatA4_h	TATTAGGGATGCAGGGCGGT		
21	S11 GatA4_h	ATGTACCTGGATGCGACGG	59	451
22	AS11 GatA4_h	GAGCAGCAGAACGTTTTGTTCTTA		
23	S12 GatA4_h	GTTCACTGCAGACCCTTCG	60	400
24	AS12 GatA4_h	TCAGAGTCGGAGGCTCCCT		
25	S13 GatA4_h	TCCTGGAAAGAAGACGACTG	55	490
26	AS13 GatA4_h	TGAGTGGGTCAGTGCCTACA		
27	S3 GatA4_h	CCTATGTCCCCTGGGTAACCTTATT	59	516
28	AS3 GatA4_h	CTGGAATGAAGCAGAGTCAGCACAC		

Supplementary Table 2: The list of transcription factors and their binding score with the wild type and mutant sequence.

Transcription factor	Sequence		Score		Strand	
	**Wild**	**Mutant**	**Wild**	**Mutant**	**Wild**	**Mutant**
**Myf1**	-	AGGCGGCTGGCG	-	7.360		+
**AP2alpha**	GCGGCCGGC	GCGGCTGGC	7.714	6.754	-	-
**AP2alpha**	CGCAGGGGC	CGCAGGGGC	5.792	5.792	-	-
**AP2alpha**	GCCGCGAGA	GCCGCGAGA	6.233	6.233	+	+
**AP2alpha**	GCGAGAGGC	GCGAGAGGC	6.540	6.540	-	-
**GATA-3**	CGAGAG	CGAGAG	4.530	4.530	+	+

Supplementary Table 3: Haplotype based association analysis of block 1 for VSD samples

Haplotype	Freq.	Case, Control Ratio Counts	Case,Control Frequencies	Chi Square	P Value
GG	0.952	54.0 : 10.0, 388.0 : 12.0	0.843, 0.970	19.589	9.6045E-6
GT	0.022	5.0 : 59.0, 5.0 : 395.0	0.079, 0.013	11.392	7.0E-4
TT	0.017	5.0 : 59.0, 3.0 : 397.0	0.078, 0.007	16.099	6.0108E-5

Supplementary Table 4: Permutation based analysis for haplotype association: 10000 permutations.

**Name**	**Chi Square**	**Permutation p-value**
Block 1: GG	19.589	0.0008
Block 1: TT	16.099	0.0029
Block 1: GT	11.392	0.0131

Supplementary Figure 1: Hardy-Weinberg equilibrium P-value.

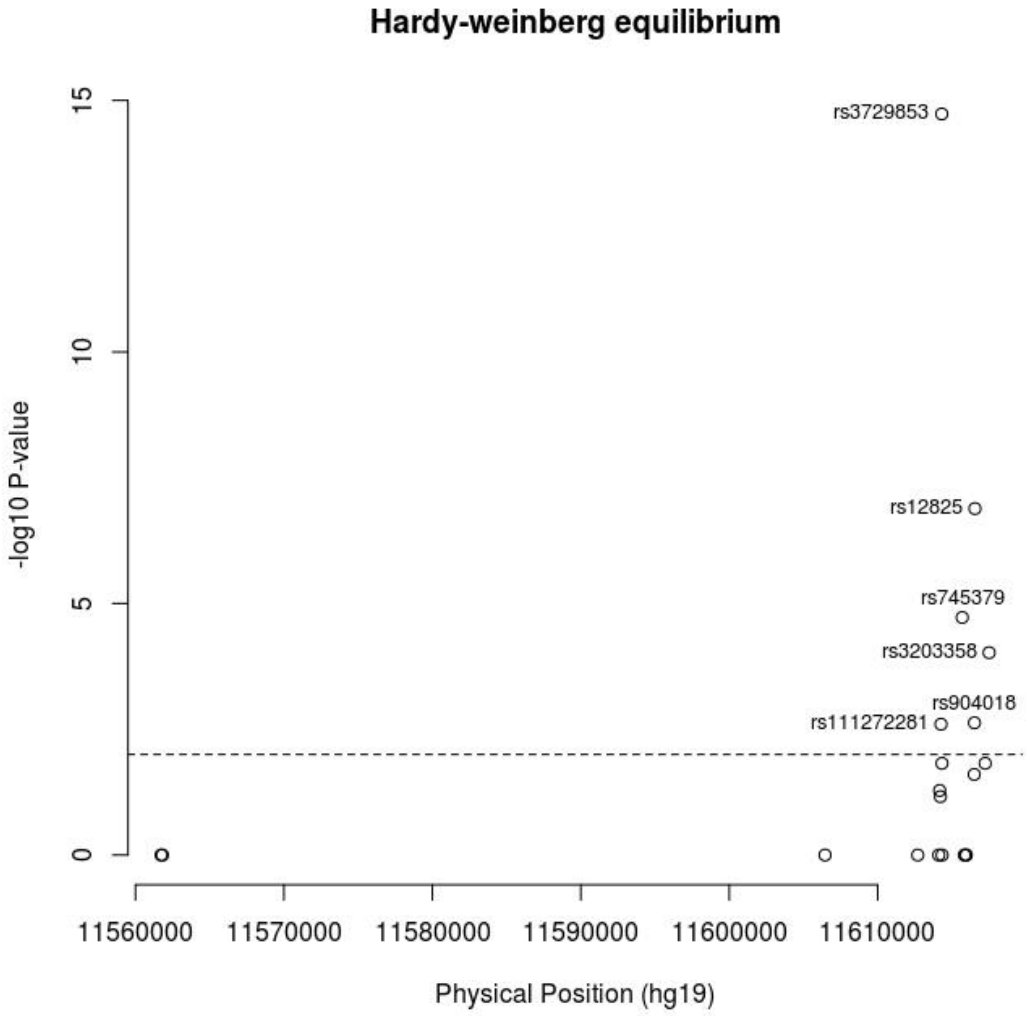

Supplementary Figure 2: Linkage disequilibrium in TOF samples

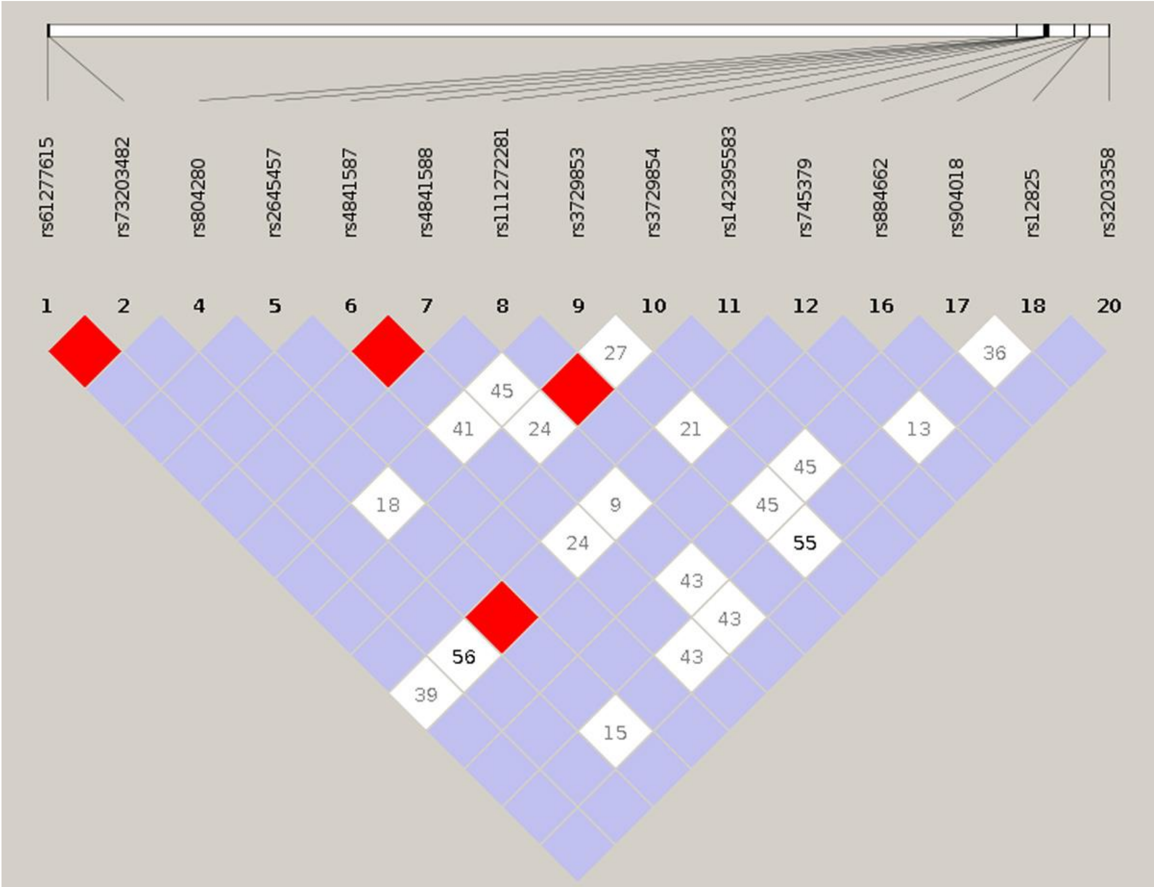

Supplementary Figure 3: Distribution of minor allele frequency of observed SNPs incontrol and 1000 genome project samples (ASN: Asian,CHB: Han Chinese in Bejing, China, CHS: Southern Han Chinese, JPT: Japanese in Tokyo, Japan, , EUR : European, AMR: American).

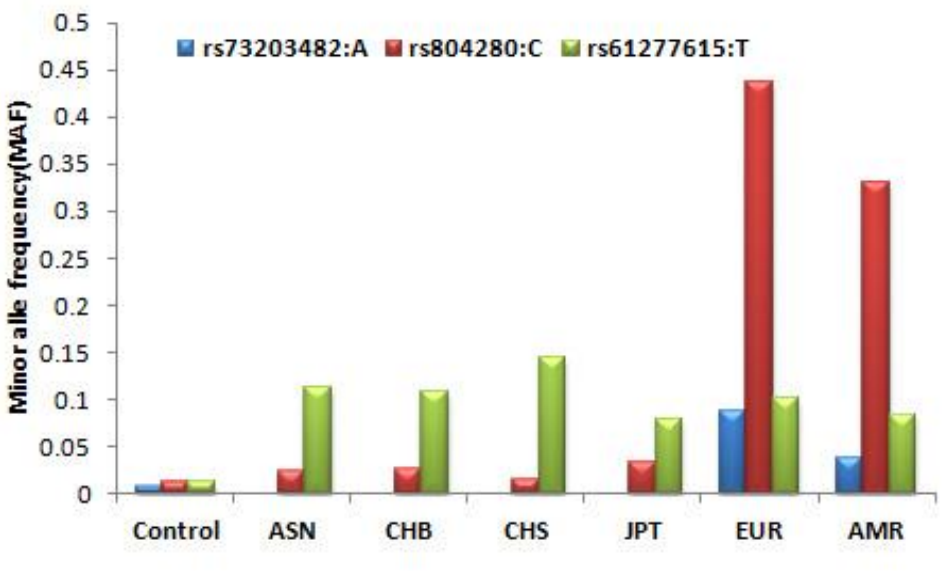

Supplementary Figure 4:

A. Free energy change (ΔΔG) for Micro RNA binding with wild-type of 3’UTR region.

B. Free energy change (ΔΔG) for Micro RNA binding with mutant of 3’UTR region.

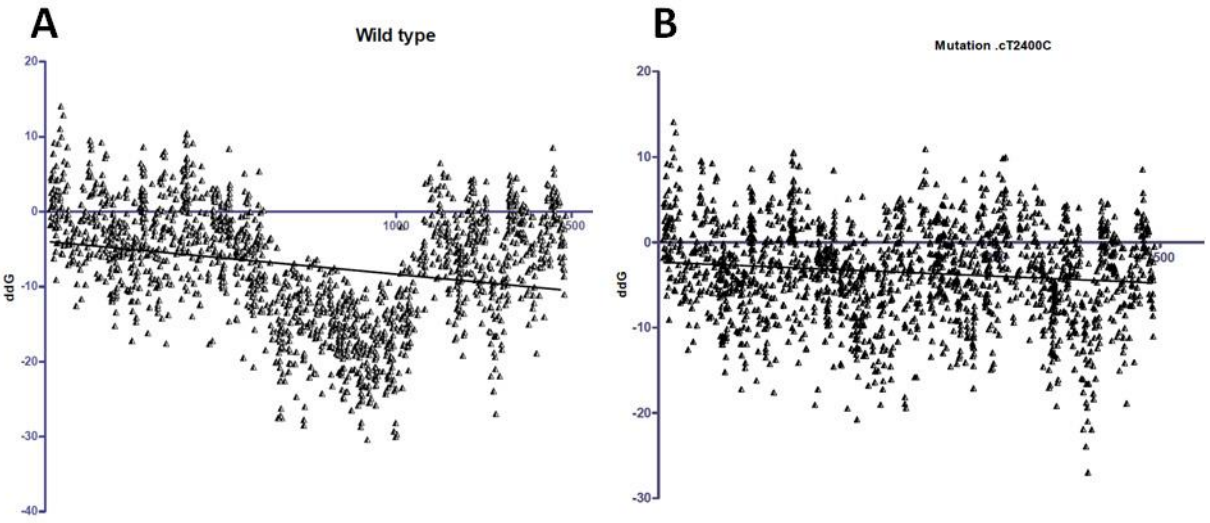

Supplementary Table (new)**:** Statistical data of *GATA4*mutations and their association with CHD (combined samples of ASD, VSD, TOF and SV).

**S.No.**	**dbSNP**	**P-value (100samples)**
2	rs73203482	0.01312
3	CM051488	0.8013
4	rs804280	0.01292
5	rs2645457	0.204
6	rs4841587	0.04872
7	rs4841588	0.0009405
8	rs111272281	0.01184
9	rs3729853	0.0417
10	rs3729854	0.03141
11	rs142395583	0.7881
12	rs745379	4.077E-10
13	rs200319078	1
14	rs56208331	0.6165
15	rs884662	0.7881
16	rs904018	0.5791
17	rs12825	0.5911
18	rs12458	0.1559
19	rs3203358	0.8013
